# Supplementary material for: Switching between H- and J-type electronic coupling in single conjugated polymer aggregates
Source: Nat Commun. 2017 Nov 21;8:1641. doi: 10.1038/s41467-017-01773-0 (PMC5696370; doi:10.1038/s41467-017-01773-0)
Supplement: Supplementary file 1 — Supplementary Information [file 41467_2017_1773_MOESM1_ESM.pdf]

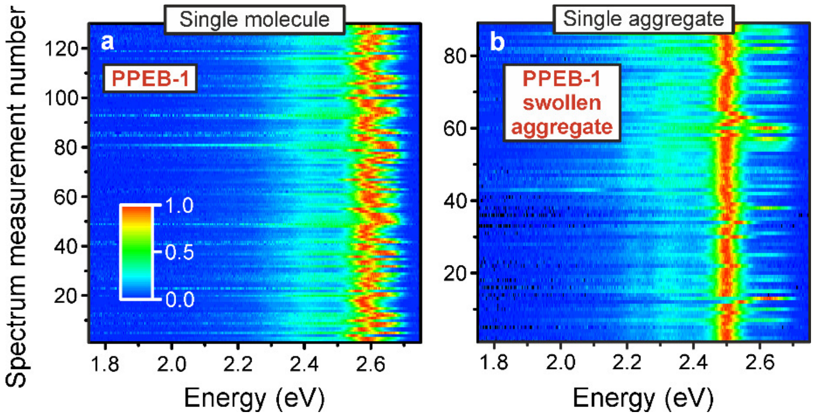

Supplementary Figure 1. (a) Normalized single-molecule spectra plotted on a false-color intensity scale of PPEB-1. (b) Single-aggregate spectra of swollen PPEB-1 aggregates. Aggregates were grown by *in situ* SVA and swollen as described in the main text.

Supplementary Note 1

Synthesis

Materials and equipment

Reagents were purchased at reagent grade from commercial sources and used without further purification. All air-sensitive reactions were carried out using standard Schlenk techniques under argon. Reaction solvents (tetrahydrofuran, dichloromethane, dimethylformamide, piperidine, pyridine) were dried, distilled, and stored under argon according to standard methods, workup solvents were purified by distillation (dichloromethane (DCM), cyclohexane (CH) and ethyl acetate (EA)). ^{1}H- and ^{13}C-NMR spectra were recorded on a Bruker DPX 400 and DRX 500 (400.1 and 500.1 MHz for ^{1}H; 100.6 and 125.8 MHz for ^{13}C). Chemical shifts are given in parts per million (ppm) referenced to residual ^{1}H or ^{13}C signals in deuterated solvents.[1] All NMR spectra were recorded at room temperature. Mass spectra were measured on a Bruker Daltronics autoflex TOF/TOF (MALDI-MS) and a Thermo Fisher Scientific Orbitrap XL (APCI). Thin layer chromatography was conducted on silica gel coated aluminium plates (Macherey-Nagel, Alugram SIL G/UV254, 0.25 mm coating with fluorescence indicator). Silica gel Kieselgel 60 (Merck, 0.040-0.063 mm) was used as the stationary phase for column chromatography.

Gel permeation chromatography (GPC) was performed in THF (analytical reagent grade, stabilized with 2.5 ppm BHT) at 30 or 35 °C. GPC analyses were run on an Agilent Technologies system at a flow rate of 1 mL/min using an IsoPump G1310 A, a diode array UV detector (G1315B) and PSS columns (Polymer Standards Service, Mainz, Germany; 10^2, 10^3, 10^5 and 10^6 Å, 5 µm, 8 x 300 mm). All molecular weights were determined versus PS calibration (PS standards from PSS, Mainz, Germany). For the preparative separation, a Shimadzu Recycling GPC (recGPC) system, equipped with a LC-20 AD pump, a SPD-20 A UV detector and a set of three preparative columns from PSS (10^3 Å, 5 µm, 20 x 300 mm) was employed. The system operated at a flow rate of 5 mL/min.

In reactions in which dry air was used, air was directed through a 20 cm column filled with $CaCl_2$.

PPEB-1

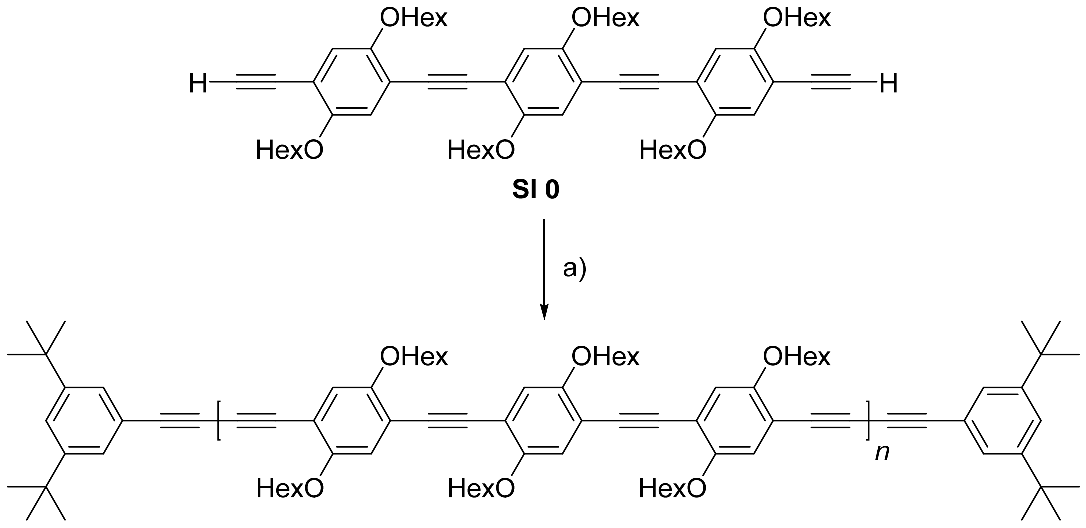

Scheme 1: Synthetic pathway towards **PPEB-1**. Compound **SI 0** was synthesized according to JESTER *et al.*[2] a) 1. TMEDA, CuCl, air, 1,2-dichlorobenzene, 35 °C, 4 h; 2. 1,3-Di-*tert*-butyl-5-ethinylbenzene[3], 35 °C, 2 h.

PPEB-1

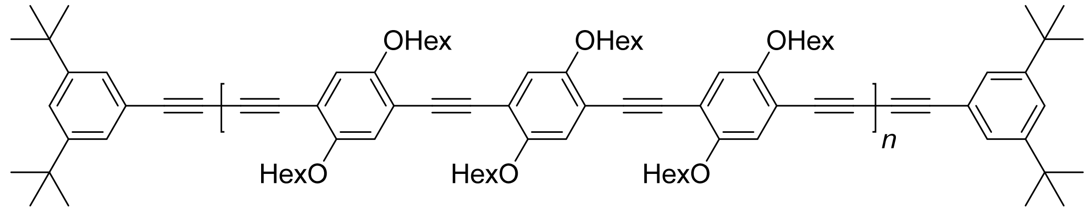

CuCl (4.5 mg, 4.5 µmol) and TMEDA (50 µL, 33 µmol) were dissolved in 1,2-dichlorobenzene (1 mL). A solution of **SI 0** (30 mg, 32 µmol) in 1,2-dichlorobenzene (3 mL) was added. The resulting mixture was stirred for 4 h at 35 °C and purged with dry air. 1,3-Di-*tert*-butyl-5-ethinylbenzene[3] (27 mg, 124 µmol) was added. The reaction mixture was stirred for 2 h at 35 °C and purged with dry air. The reaction mixture was diluted with water. The aqueous phase was extracted with DCM and the combined organic phases were washed with aqueous NH_3 (25%), water and brine and dried over $MgSO_4$. The product was precipitated from DCM with MeOH to give 20 mg of **PPEB-1** as an orange solid. The product was separated into 4 fractions with low polydispersity via recGPC, from which one sample was investigated in detail ($M_n = 40.0 \cdot 10^3$ g mol^{-1}, $M_p = 50.5 \cdot 10^3$ g mol^{-1}, $M_w = 58.4 \cdot 10^3$ g mol^{-1}, $PD = 1.46$).

Chemical formula: $C_{62}H_{84}O_6$ (repeating unit), $C_{94}H_{126}O_6$ (n=1), molecular weight: 925.35 g mol^{-1} (repeating unit), 1352.04 g mol^{-1} (n=1).

^{1}H-NMR (400 MHz, $CDCl_3$): δ [ppm] = 7.01 (s, 2H), 6.99 (s, 4H), 4.08-3.96 (m, 12H), 1.91-1.76 (m, 12H), 1.58-1.47 (m, 30H), 1.43-1.30 (m, 24H), 0.97-0.85 (m, 18H).

GPC (PS-calibration in THF): $M_n = 25.4\cdot10^3$ g mol^{-1}, $M_p = 61.3\cdot10^3$ g mol^{-1}, $M_w = 67.3\cdot10^3$ g mol^{-1}, $PD = 2.65$.

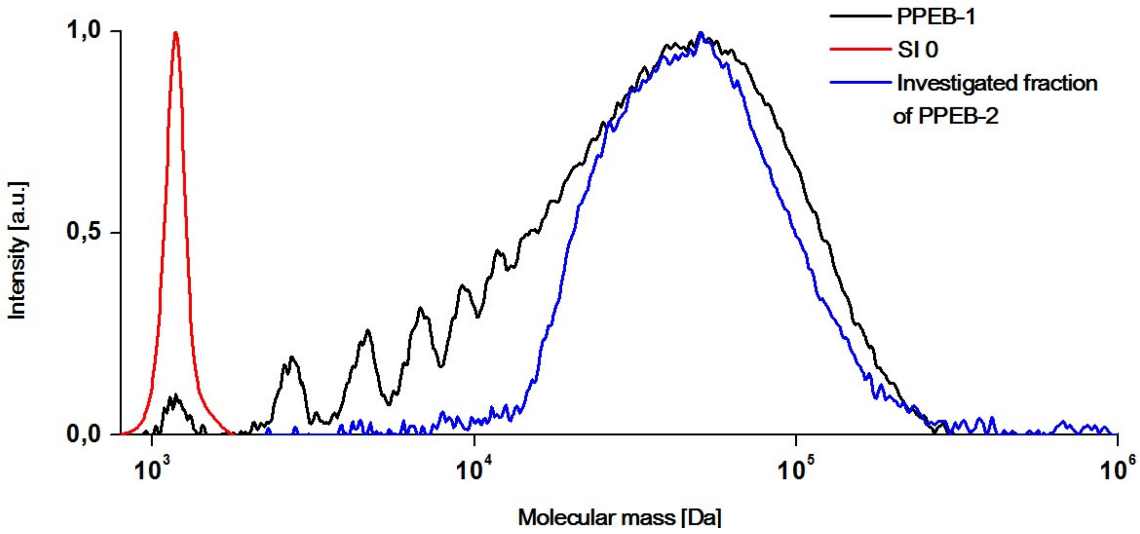

Supplementary Figure 2: GPC elugram of **PPEB-1** (black): $M_n = 25.4\cdot10^3$ g mol^{-1}, $M_p = 61.3\cdot10^3$ g mol^{-1}, $M_w = 67.3\cdot10^3$ g mol^{-1}, $PD = 2.65$; **SI 0** (red): $M_n = 1.2\cdot10^3$ g mol^{-1}; $M_p = 1.2\cdot10^3$ g mol^{-1}, $M_w = 1.2\cdot10^3$ g mol^{-1}, $PD = 1.01$; **investigated fraction of PPEB-1** (blue): $M_n = 40.0\cdot10^3$ g mol^{-1}, $M_p = 50.5\cdot10^3$ g mol^{-1}, $M_w = 58.4\cdot10^3$ g mol^{-1}, $PD = 1.46$.

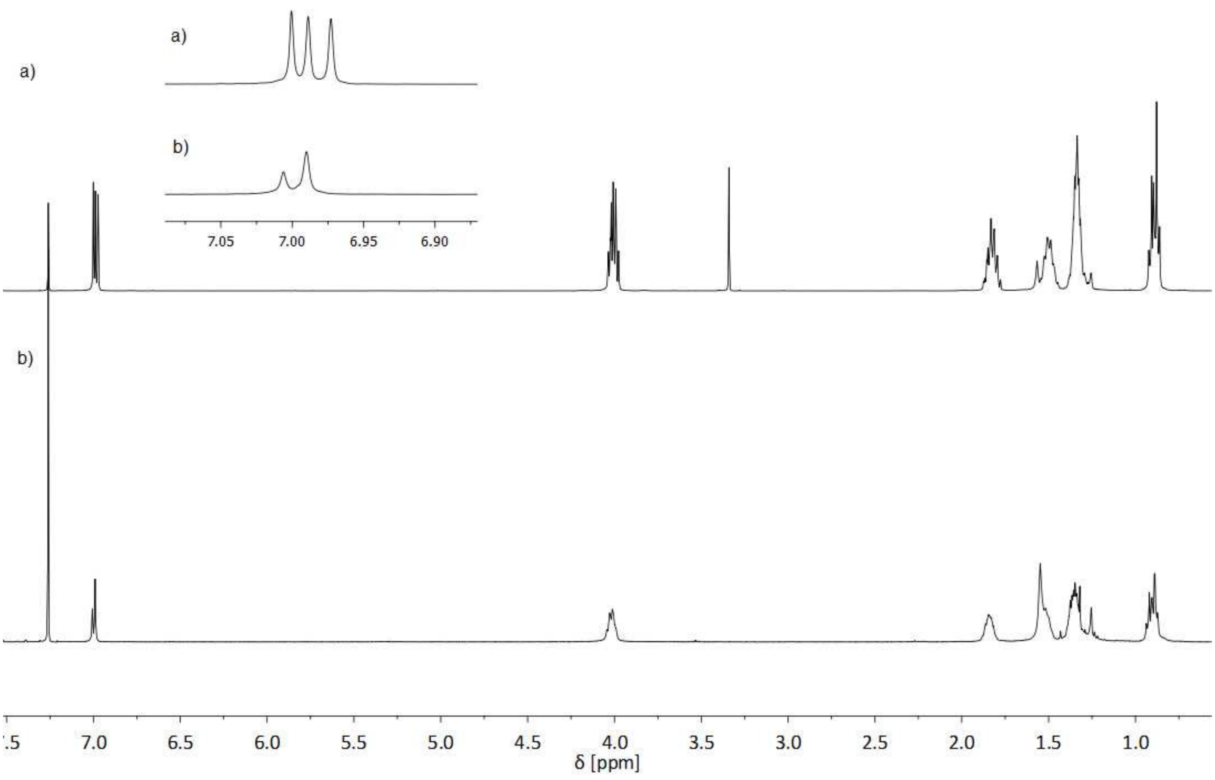

Supplementary Figure 3: a) ^{1}H-NMR spectrum (400 MHz, $CDCl_3$) of **SI 0**; b) ^{1}H-NMR spectrum (400 MHz, $CDCl_3$) of **PPEB-1**.

PPEB-2

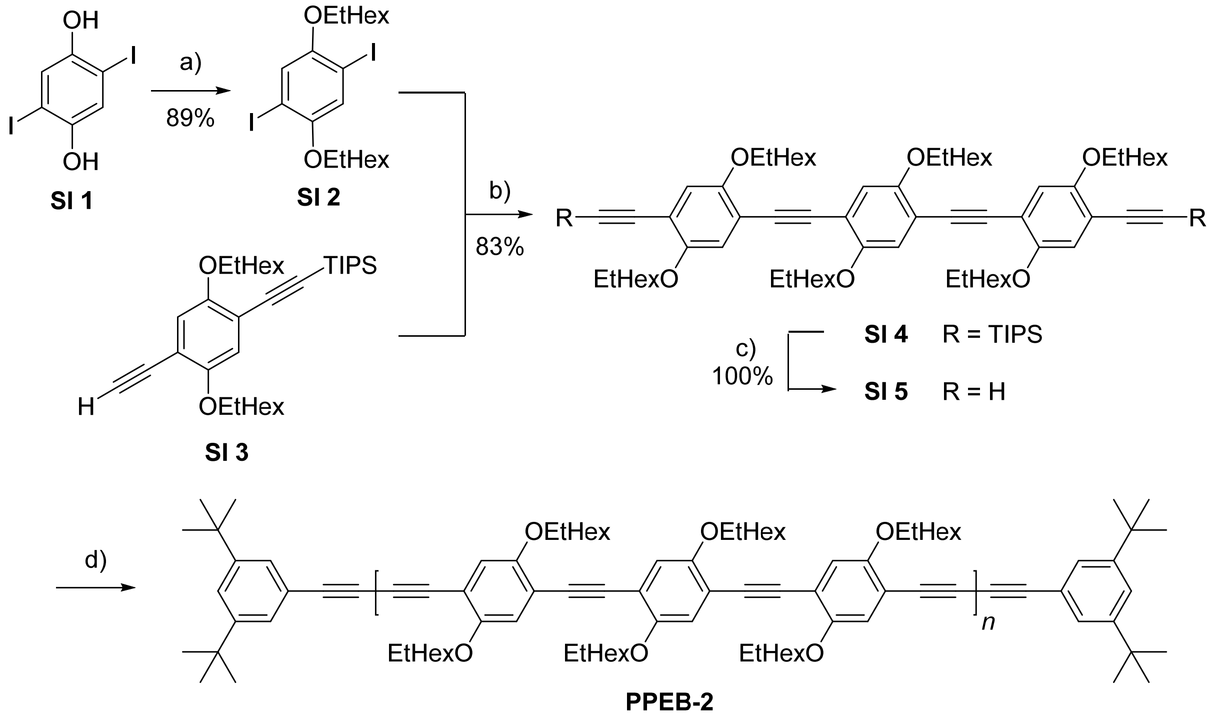

Scheme 2: Synthetic pathway towards **PPEB-2**. Compounds **SI 1** and **SI 3** were synthesized according to WATANABE *et al.*[4], JESTER *et al.* respectively.[5] Endcaps (1,3-di-*tert*-butyl-5-ethinylbenzene) were synthesized comparable to ZHANG *et al.*[3] a) K_2CO_3, 2-ethylhexyl bromide, DMF, 70 °C, 44 h; b) $Pd(PPh_3)_2Cl_2$, CuI, PPh_3, **SI 3**, piperidine, THF, 16 h, 50 °C; c) TBAF, THF, r.t., 2 h; d) 1. TMEDA, CuCl, air, 1,2-dichlorobenzene, r.t. → 40 °C, 8.5 h; 2. 1,3-Di-*tert*-butyl-5-ethinylbenzene, r.t. → 40 °C, 20 h.

SI 2

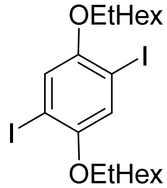

2,5-Diiodohydroquinone[4] (0.40 g, 1.10 mmol) and K_2CO_3 (0.91 g, 6.60 mmol) were suspended in DMF (15 mL) under Ar. 2-Ethylhexyl bromide (0.98 mL, 5.50 mmol) was added and the resulting reaction mixture was stirred for 44 h at 70 °C and then diluted with water. The aqueous phase was extracted with Et_2O and the combined organic phases were washed with water, aqueous 2M HCl, water and brine and dried over $MgSO_4$. After removal of the solvent, the crude product was purified by column chromatography (CH, $R_f = 0.41$) to give 0.58 g of **SI 2** as a colorless oil (89%).

Chemical formula: $C_{22}H_{36}I_2O_2$, molecular weight: 586.34 g mol^{-1}.

^{1}H-NMR (500 MHz, $CDCl_3$): δ [ppm] = 7.16 (s, 2H), 3.82 (d, ^3J = 5.4 Hz, 4H), 1.73 (sep, ^3J = 6.2 Hz, 2H), 1.62-1.38 (m, 8H), 1.38-1.28 (m, 8H), 0.97-0.88 (m, 12H).

^{13}C-NMR (126 MHz, $CDCl_3$): δ [ppm] = 153.0, 122.5, 86.2, 72.5, 39.6, 30.7, 29.2, 24.1, 23.2, 14.3, 11.4.

MS (MALDI TOF, DCTB): *m/z* (%) = 586.1 (100) $[M]^{+\bullet}$.

SI 4

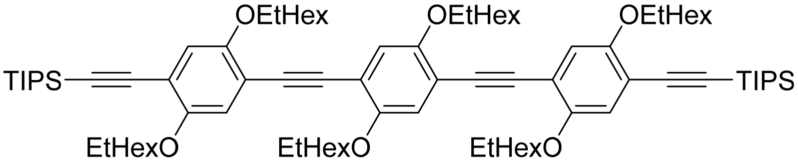

SI 2 (60 mg, 102 µmol), CuI (2.0 mg, 11 µmol), PPh_3 (3.5 mg, 13 µmol) and $Pd(PPh_3)_2Cl_2$ (3.5 mg, 5 µmol) were dissolved in dry piperidine (5 mL) under Ar. **SI 3**[5] (110 mg, 204 µmol) and dry THF (2mL) were added. The resulting reaction mixture was stirred for 16 h at 50 °C and then diluted with water. The aqueous phase was extracted with DCM and the combined organic phases were washed with aqueous 2M HCl, water and brine and dried over $MgSO_4$. After removal of the solvent, the crude product was purified by column chromatography (CH:DCM 5:1 → CH:DCM 5:2, R_f = 0.18 (5:1), 0.40 (5:2)) to give 119 mg of **SI 4** as a yellow oil (83%).

Chemical formula: $C_{92}H_{150}O_6Si_2$, molecular weight: 1408.38 g mol^{-1}.

^{1}H-NMR (500 MHz, CD_2Cl_2): δ [ppm] = 6.98 (s, 2H), 6.95 (s, 2H), 6.94 (s, 2H), 3.96-3.79 (m, 12H), 1.84-1.69 (m, 6H), 1.68-1.24 (m, 54H), 1.15 (s, 36H), 1.00-0.86 (m, 36H).

^{13}C-NMR (126 MHz, CD_2Cl_2): δ [ppm] = 154.8, 154.2, 153.9, 118.0, 117.0, 116.4, 114.7, 114.5, 114.2,103.6, 96.9, 92.1, 91.9, 72.5, 72.3, 71.9, 40.2, 40.2, 31.2, 31.1, 30.8, 29.7, 29.6, 29.6, 24.6, 24.2, 23.7,19.0, 14.4, 14.4, 11.9, 11.7, 11.6, 11.4.

MS (MALDI TOF, DCTB): *m/z* (%) = 1407.1 (100) $[M]^{+\bullet}$.

SI 5

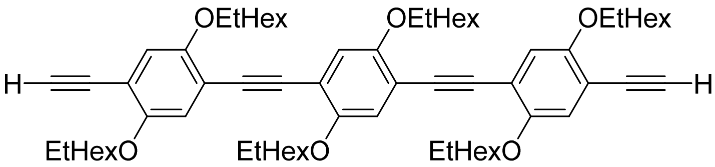

SI 4 (101 mg, 72 µmol) was dissolved in dry THF (8 mL) under Ar and a solution of TBAF in THF (1M, 180 µL, 180 µmol) was added dropwise. The resulting reaction mixture was stirred for 2 h and then diluted with water. The aqueous phase was extracted with DCM and the combined organic phases were washed with water and brine and dried over $MgSO_4$. After removal of the solvent, the crude product was purified by column chromatography (CH:DCM 5:2, R_f = 0.54) and recGPC to give 67 mg of **SI 5** as a yellow oil (85%).

Chemical formula: $C_{74}H_{110}O_6$, molecular weight: 1095.69 g mol^{-1}.

^{1}H-NMR (400 MHz, $CDCl_3$): δ [ppm] = 6.99 (s, 2H), 6.98 (s, 2H), 6.97 (s, 2H), 3.95-3.83 (m, 12H), 3.32 (s, 2H), 1.86-1.72 (m, 6H), 1.68-1.24 (m, 48H), 1.04-0.80 (m, 36H).

^{13}C-NMR (126 MHz, $CDCl_3$): δ [ppm] = 154.6, 153.9, 153.7, 117.7, 116.8, 116.8, 115.1, 114.3, 112.6, 91.7, 91.4, 82.3, 80.2, 72.2, 72.1 72.0, 39.7, 39.7, 39.6, 30.8, 30.7, 29.3, 29.2, 24.2, 24.0, 23.2, 23.2, 14.2, 14.2, 11.5, 11.4, 11.3.

MS (MALDI TOF, DCTB): *m/z* (%) = 1094.8 (100) $[M]^{+\bullet}$.

GPC (PS-calibrated in THF): $M_p = 1.3 \cdot 10^3$ g mol^{-1}.

PPEB-2

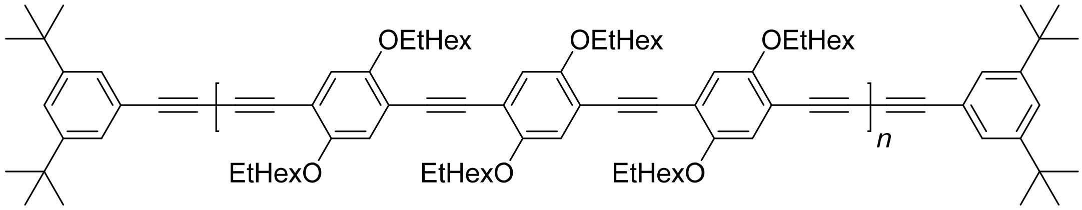

SI 5 (67 mg, 61 µmol) and TMEDA (92 µL, 609 µmol) were dissolved in 1,2-dichlorobenzene (7 mL) and purged with dry air. After 10 min CuCl (8.4 mg, 85 µmol) was added. The resulting mixture was stirred for 2.5 h at r.t., 30 min at 38 °C, 70 min at r.t., 30 min at 38 °C, 2 h at r.t., 60 min at 40 °C and 60 min at r.t. while purging with dry air continued. 1,3-Di-*tert*-butyl-5-ethinylbenzene[3] (51 mg, 236 µmol) was added. The reaction mixture was stirred for 19 h at

r.t. and then 60 min at 40 °C. During the last 60 min, dry air was purged through the solution. The reaction mixture was diluted with water. The aqueous phase was extracted with DCM and the combined organic phases were washed with aqueous NH_3 (25%), water and brine and dried over $MgSO_4$. The product was precipitated from DCM with MeOH to give 48 mg of **PPEB-2** as an orange solid. The product was separated into 15 fractions with low polydispersity via recGPC, from which one sample was investigated in detail ($M_n = 66.0 \cdot 10^3$ g mol^{-1}, $M_p = 66.2 \cdot 10^3$ g mol^{-1}, $M_w = 69.4 \cdot 10^3$ g mol^{-1}, $PD = 1.05$).

Chemical formula: $C_{74}H_{108}O_6$ (repeating unit), $C_{106}H_{150}O_6$ (n=1), molecular weight: 1093.67 g mol^{-1} (repeating unit), 1520.36 g mol^{-1} (n=1).

^{1}H-NMR (400 MHz, $CDCl_3$): δ [ppm] = 6.98 (br s, 4H), 6.97 (br s, 2H), 3.96-3.83 (m, 12H), 1.85-1.74 (m, 6H), 1.69-1.20 (m, 48H), 1.01-0.80 (m, 36H).

GPC (PS-calibration in THF): $M_n = 20.1 \cdot 10^3$ g mol^{-1}, $M_p = 45.9 \cdot 10^3$ g mol^{-1}, $M_w = 57.6 \cdot 10^3$ g mol^{-1}, $PD = 2.87$.

Supplementary Table 1: GPC peak molecular weights, calculated molecular weights and mass peaks of **PPEB-2**.

Repeating unit n	GPC (PS-calibration) M_p (g mol^{-1})	calculation M (g mol^{-1})	MS (MALDI TOF, DCTB) m/z (%)
1	$2.0 \cdot 10^3$	1520.3	1519.0 (100) $[M]^{+\bullet}$
2	$3.5 \cdot 10^3$	2614.0	2613.3 (100) $[M]^{+\bullet}$
3	$5.7 \cdot 10^3$	3707.6	3707.2 (100) $[M]^{+\bullet}$
4	$7.9 \cdot 10^3$	4801.3	4801.6 (61) $[M]^{+\bullet}$
5	$10.6 \cdot 10^3$	5895.0	5894.9 (100) $[M]^{+\bullet}$
6	$13.3 \cdot 10^3$	6988.6	7236.0 (100) $[M+DCTB]^{+\bullet}$

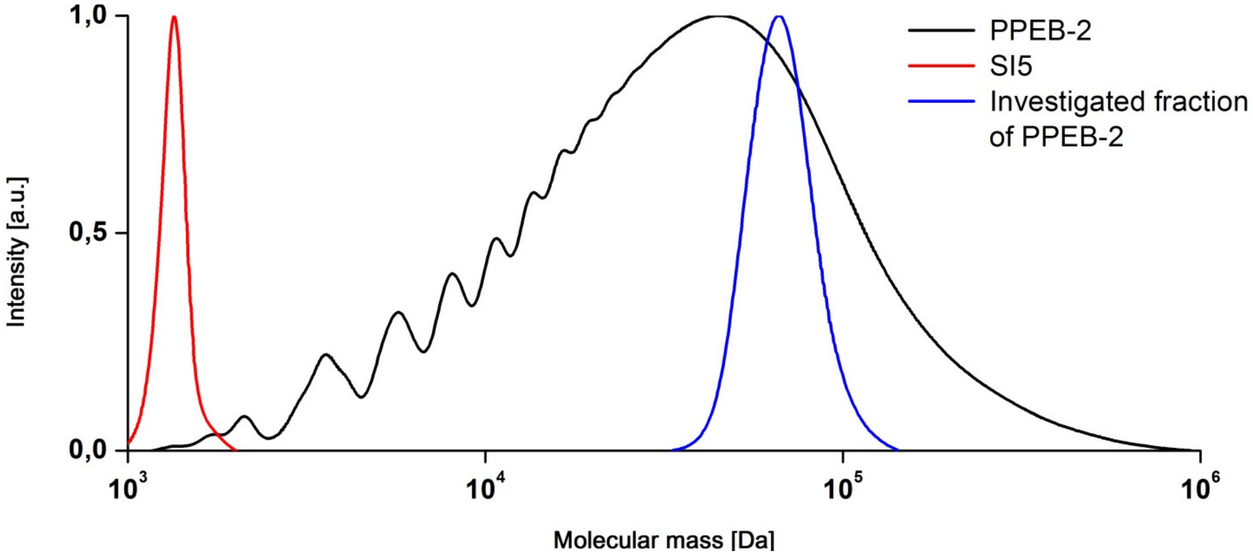

Supplementary Figure 4: GPC elugram of **PPEB-2** (black): $M_n = 20.1\cdot 10^3$ g mol^{-1}, $M_p = 45.9\cdot 10^3$ g mol^{-1}, $M_w = 57.6\cdot 10^3$ g mol^{-1}, $PD = 2.87$; **SI5** (red): $M_n = 1.3\cdot 10^3$ g mol^{-1}; $M_p = 1.3\cdot 10^3$ g mol^{-1}, $M_w = 1.3\cdot 10^3$ g mol^{-1}, $PD = 1.01$; **investigated fraction of PPEB-2** (blue): $M_n = 66.0\cdot 10^3$ g mol^{-1}, $M_p = 66.2\cdot 10^3$ g mol^{-1}, $M_w = 69.4\cdot 10^3$ g mol^{-1}, $PD = 1.05$.

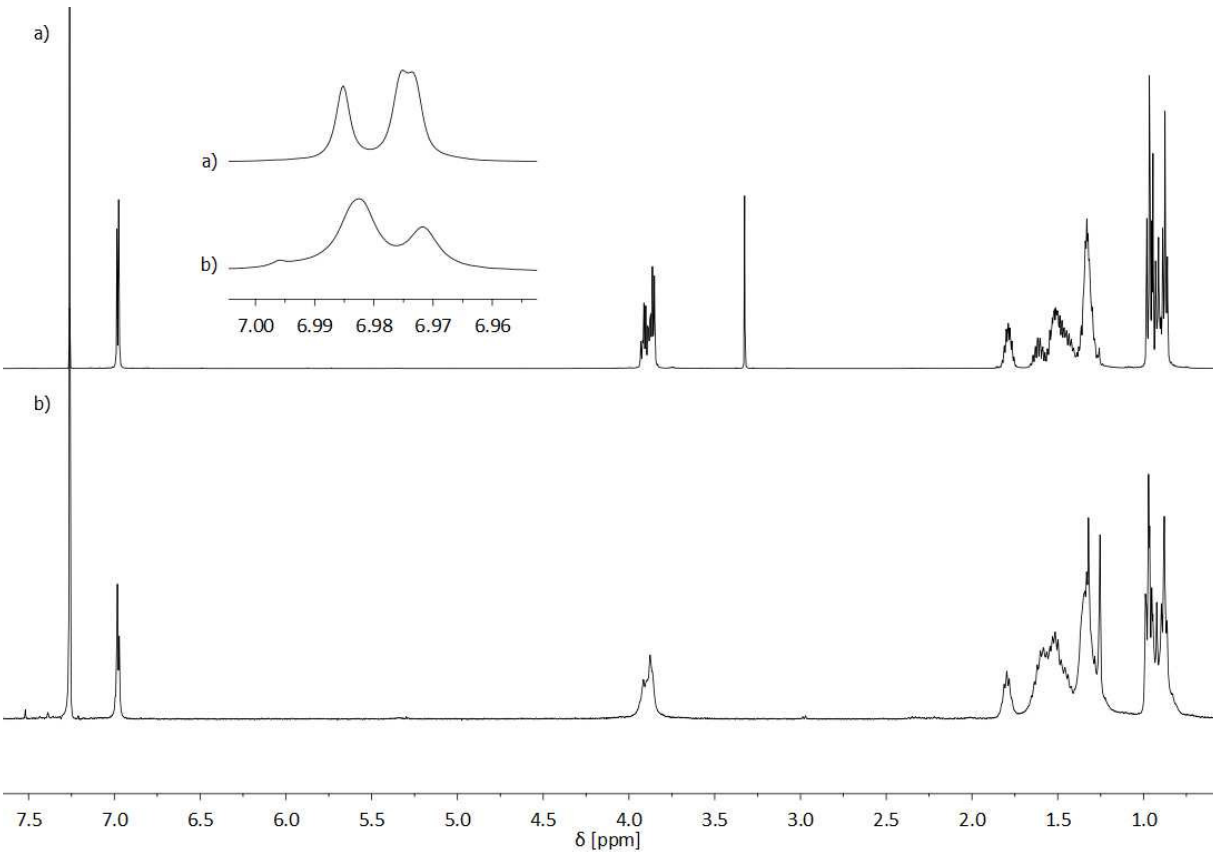

Supplementary Figure 5: a) ^{1}H-NMR spectrum (500 MHz, $CDCl_3$) of **SI 5**; b) ^{1}H-NMR spectrum (400 MHz, $CDCl_3$) of **PPEB-2**.

PPEB-3

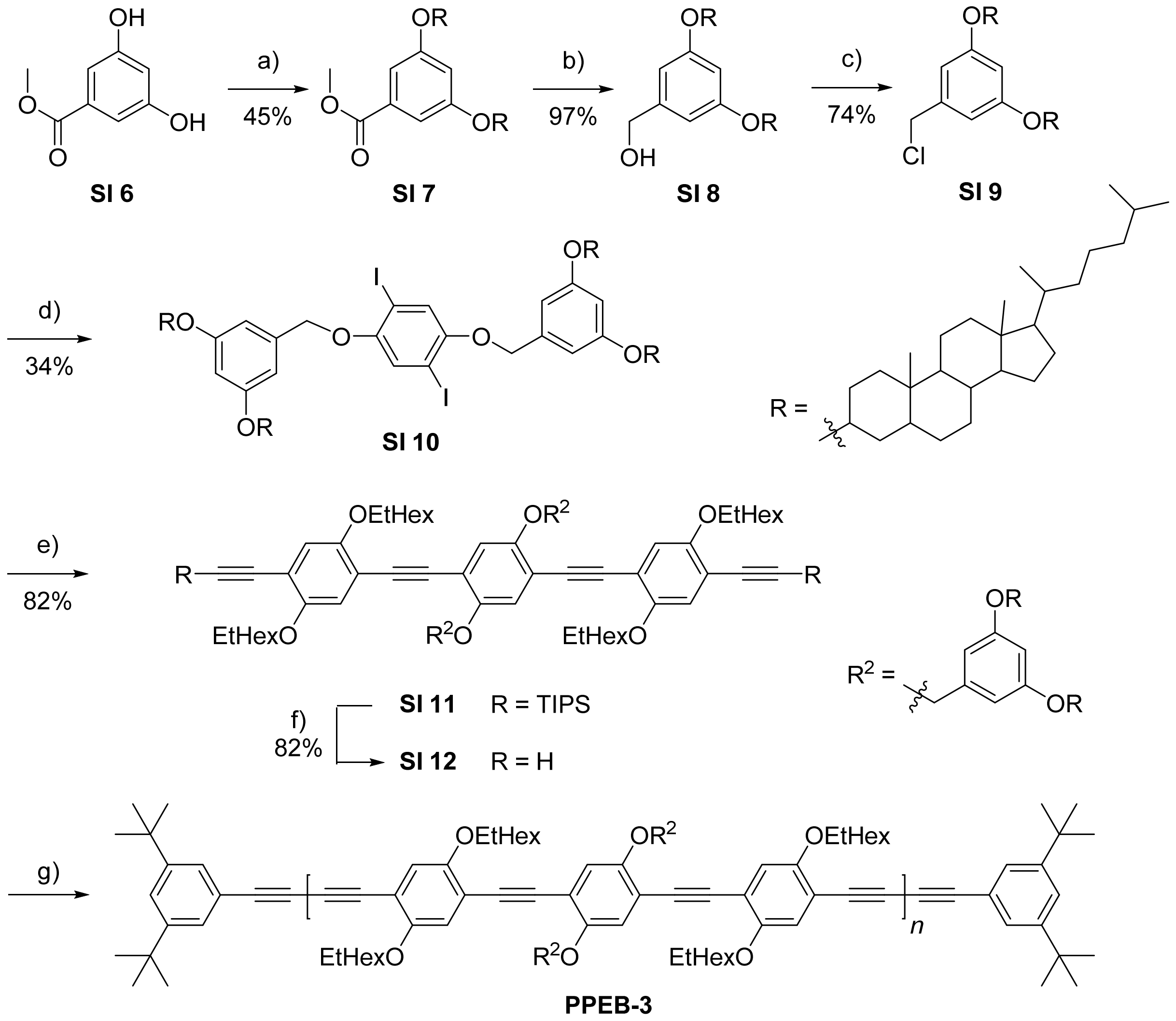

Scheme 3: Synthetic pathway towards **PPEB-3**. a) Cholestanol, PPh_3, DIAD, THF, 0 °C → r.t., 22 h; b) DIBAL, THF, 0 °C → r.t., 2.5 h; c) $SOCl_2$, pyridine, DCM, 0 °C → r.t., 3.5 h; d) 2,5-diiodohydroquinone[4], K_2CO_3, KI, DMF, 80 °C, 2 d; e) $Pd(PPh_3)_2Cl_2$, CuI, PPh_3, **SI 3**[5], piperidine, 50 °C, 16 h; f) TBAF, THF, r.t., 6.5 h; g) 1. CuCl, $CuCl_2$, pyridine, r.t. → 50 °C, 7 h; 2. 1,3-Di-*tert*-butyl-5-ethinylbenzene[3], 50 °C, 15 h.

SI 7

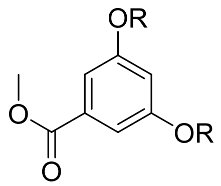

Methyl-3,5-dihydroxybenzoate (1.50 g, 8.92 mmol), cholestanol (7.63 g, 19.6 mmol) and PPh_3 (5.15 g, 19.6 mmol) were dissolved in dry THF (70 mL) under Ar. The reaction mixture was cooled to 0 °C and diisopropyl azodicarboxylate (3.97 g, 19.6 mmol) in 5 mL dry DCM was

added dropwise. The reaction mixture was allowed to reach r.t. and stirring was continued for 22 h. The mixture was then diluted with aqueous 1M NaOH solution and Et_2O. The resulting precipitate was filtered and washed with Et_2O and the organic phase was dried over $MgSO_4$. After removal of the solvent, the crude product was purified by column chromatography (CH:EA 60:1, R_f = 0.20) to give 3.65 g of **SI 7** as a white solid (45%).

Chemical formula: $C_{62}H_{100}O_4$, molecular weight: 909.46 g mol^{-1}.

^{1}H-NMR (500 MHz, CD_2Cl_2): δ [ppm] = 7.10 (d, ^4J = 2.3 Hz, 2H), 6.64 (t, ^4J = 2.3 Hz, 1H), 4.58-4.54 (m, 2H), 3.85 (s, 3H), 2.01-1.95 (m, 2H), 1.89-1.77 (m, 4H), 1.72-1.45 (m, 18H), 1.42-0.93 (m, 36H), 0.91(d, ^3J = 6.5 Hz, 6H), 0.86 (dd, ^3J = 6.5 Hz, ^4J = 2.1 Hz, 12H), 0.83 (s, 6H), 0.82-0.75 (m, 2H), 0.67 (s, 6H).

^{13}C-NMR (126 MHz, CD_2Cl_2): δ [ppm] = 167.4, 159.5, 132.6, 109.8, 109.4, 73.1, 57.2, 56.9, 54.8, 52.5,43.1, 40.7, 40.2, 40.1, 36.7, 36.4, 36.3, 36.1, 33.2, 33.1, 32.6, 29.1, 28.8, 28.6, 26.2, 24.7, 24.4, 23.1,22.9, 21.4, 19.0, 12.4, 11.8.

MS (MALDI TOF, DCTB): *m/z* (%) = 908.8 (34) $[M]^{+\bullet}$, 931.9 (53) $[M+Na]^{+\bullet}$.

SI 8

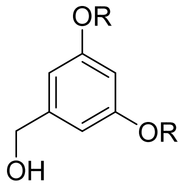

SI 7 (3.27 g, 3.60 mmol) was dissolved in dry THF (50 mL) under Ar. The reaction mixture was cooled to 0 °C and a DIBAL solution (1M in *n*-hexane, 9.00 mL, 9.00 mmol) was added dropwise over 5 min. The reaction mixture was allowed to reach r.t. and stirring was continued for 2.5 h. Ice was added and the aqueous phase was extracted with MTBE. The combined organic phases were washed with water and brine and dried over $MgSO_4$. Evaporation of the solvent under reduced pressure yielded 3.06 g of **SI 8** as a white solid (97%).

Chemical formula: $C_{61}H_{100}O_3$, molecular weight: 881.44 g mol^{-1}.

^{1}H-NMR (500 MHz, $CDCl_3$): δ [ppm] = 6.48 (d, ^4J = 2.1 Hz, 2H), 6.39 (t, ^4J = 2.1 Hz, 1H), 4.60 (s, 2H), 4.51 (s, 2H), 2.00-1.94 (m, 2H), 1.92-1.76 (m, 4H), 1.69-1.42 (m, 18H), 1.41-0.95

(m, 36H), 0.90 (d,^3J = 6.5 Hz, 6H), 0.86 (dd, ^3J = 6.5 Hz, ^4J = 2.4 Hz, 12H), 0.81 (s, 6H), 0.79-0.73 (m, 2H), 0.65 (s, 6H).

^{13}C-NMR (126 MHz, $CDCl_3$): δ [ppm] = 159.4, 143.2, 106.5, 103.5, 72.2, 65.7, 56.7, 56.4, 54.3, 42.7,40.2, 39.7, 39.7, 36.3, 36.0, 36.0, 35.7, 32.9, 32.8, 32.1, 28.6, 28.4, 28.2, 25.9, 24.3, 24.0, 23.0, 22.7,21.0, 18.8, 12.2, 11.6.

MS (MALDI TOF, DCTB): *m/z* (%) = 880.8 (81) $[M]^{+\bullet}$, 903.8 (29) $[M+Na]^{+\bullet}$.

SI 9

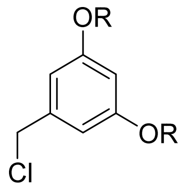

SI 8 (1.75 g, 1.99 mmol) and pyridine (160 µL, 1.99 mmol) were dissolved in dry DCM (40 mL) under Ar. The reaction mixture was cooled to 0 °C and $SOCl_2$ (145 µL, 1.99 mmol) was added. The reaction mixture was allowed to reach r.t. and stirring was continued for 3.5 h. After removal of the solvent, the crude product was purified by column chromatography (CH:DCM 9:1, R_f = 0.54) to give 1.32 g of **SI 9** as a white solid (74%).

Chemical formula: $C_{61}H_{99}ClO_2$, molecular weight: 899.89 g mol^{-1}.

^{1}H-NMR (500 MHz, $CDCl_3$): δ [ppm] = 6.49 (d, ^4J = 2.1 Hz, 2H), 6.40 (t, ^4J = 2.1 Hz, 1H), 4.52 (s, 2H), 4.49 (s, 2H), 2.00-1.94 (m, 2H), 1.92-1.76 (m, 4H), 1.69-1.44 (m, 18H), 1.40-0.94 (m, 36H), 0.90 (d,^3J = 6.5 Hz, 6H), 0.87 (dd, ^3J = 6.6 Hz, ^4J = 2.4 Hz, 12H), 0.82 (s, 6H), 0.80-0.73 (m, 2H), 0.65 (s, 6H).

^{13}C-NMR (126 MHz, $CDCl_3$): δ [ppm] = 159.3, 139.4, 108.2, 104.1, 72.3, 56.7, 56.4, 54.3, 46.7, 42.7,40.2, 39.7, 39.7, 36.3, 36.0, 35.9, 35.7, 32.9, 32.8, 32.1, 28.6, 28.4, 28.2, 25.9, 24.3, 24.0, 23.0, 22.7,21.0, 18.8, 12.2, 11.6.

MS (APCI): *m/z* (%) = 899.740 (100) $[M+H]^+$.

SI 10

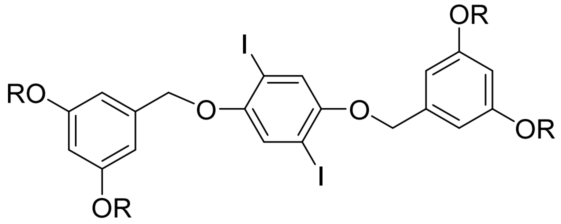

2,5-Diiodohydroquinone[4] (0.20 g, 0.56 mmol), **SI 9** (1.00 g, 1.11 mmol), K_2CO_3 (0.46 g, 3.33 mmol) and KI (37 mg, 0.22 mmol) were suspended in dry DMF. The resulting suspension was stirred at 80 °C for 2 d and then diluted with water. The aqueous phase was extracted with Et_2O and the combined organic phases were washed with water, aqueous 2M HCl, water and brine and dried over $MgSO_4$. After removal of the solvent, the crude product was purified by column chromatography (CH:DCM 6:1 → DCM, R_f = 0.02 (6:1)) to give 397 mg of **SI 10** as a white solid (34%).

Chemical formula: $C_{128}H_{200}I_2O_6$, molecular weight: 2088.81 g mol^{-1}.

^{1}H-NMR (400 MHz, $CDCl_3$): δ [ppm] = 7.25 (s, 2H), 6.61 (d, ^4J = 2.1 Hz, 4H), 6.43 (t, ^4J = 2.1 Hz, 2H), 4.97 (s, 4H) 4.53 (s, 4H), 2.01-1.47 (m, 48H), 1.41-0.94 (m, 72H), 0.91 (d, ^3J = 6.4 Hz, 12H), 0.87 (dd,^3J = 6.4 Hz, ^4J = 2.0 Hz, 24H), 0.82 (s, 12H), 0.80-0.74 (m, 4H), 0.66 (s, 12H).

^{13}C-NMR (126 MHz, $CDCl_3$): δ [ppm] = 159.3, 152.9, 138.3, 123.7, 106.8, 104.6, 86.7, 72.5, 72.2, 56.7,56.5, 54.4, 42.8, 40.2, 39.8, 39.7, 36.4, 36.0, 36.0, 35.7, 32.9, 32.9, 32.1, 30.3, 28.7, 28.4, 28.2, 25.9,24.4, 24.0, 23.0, 22.7, 21.0, 18.8, 12.3, 11.6.

MS (MALDI TOF, DCTB): *m/z* (%) = 2110.3 (65) $[M+Na]^{+\bullet}$, 2126.3 (100) $[M+K]^{+\bullet}$.

SI 11

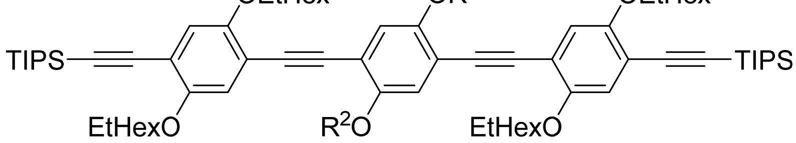

SI 10 (200 mg, 96 µmol), $Pd(PPh_3)_2Cl_2$ (6.7 mg, 9.5 µmol), PPh_3 (5.0 mg, 19.1 µmol) and CuI (3.6 mg, 18.9 µmol) were dissolved in dry piperidine (20 mL) under Ar. **SI 3**[5] (109 mg, 202 µmol) was added and the resulting mixture was stirred at 50 °C for 16 h. The reaction

mixture was diluted with water and the aqueous phase was extracted with DCM. The combined organic phases were washed with aqueous 2M HCl, water and brine and dried over $MgSO_4$. After removal of the solvent, the crude product was purified by column chromatography (CH:DCM 5:1 → CH:DCM 5:2, R_f = 0.19 (5:1), 0.53 (5:2)) to give 229 mg of **SI 11** as a yellow solid (82%).

Chemical formula: $C_{198}H_{314}O_{10}Si_2$, molecular weight: 2910.78 g mol^{-1}.

^{1}H-NMR (400 MHz, $CDCl_3$): δ [ppm] = 7.08 (s, 2H), 6.92 (s, 2H), 6.91 (s, 2H), 6.62 (d, ^4J = 2.0 Hz, 4H), 6.36 (t, ^4J = 2.0 Hz, 2H), 5.07 (s, 4H), 4.39 (s, 4H), 3.93-3.71 (m, 12H), 1.99-1.91 (m, 6H), 1.89-0.57 (m, 276H).

^{13}C-NMR (126 MHz, $CDCl_3$): δ [ppm] = 159.3, 154.6, 153.6, 153.6, 139.2, 117.9, 117.9, 116.0, 115.0,114.2, 114.1, 106.0, 104.4, 103.2, 96.3, 91.9, 91.0, 72.3, 72.3, 71.4, 71.4, 56.7, 56.5, 54.3, 42.7,40.2, 39.8, 39.7, 39.7, 36.4, 36.0, 35.9, 35.7, 32.8, 32.1, 30.8, 30.5, 30.3, 29.9, 29.3, 28.6, 28.4, 28.2,25.8, 24.3, 24.1, 24.0, 23.8, 23.3, 23.2, 23.0, 22.7, 20.9, 18.9, 18.8, 14.3, 14.3, 12.2, 11.6, 11.4, 11.3.

MS (MALDI TOF): *m/z* (%) = 2908.4 (40) $[M]^{+\bullet}$.

SI 12

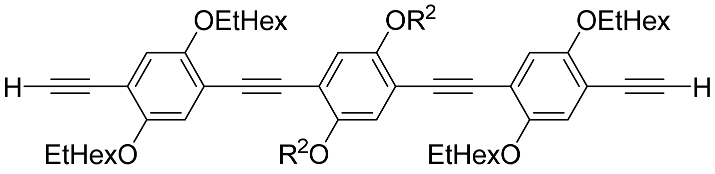

SI 11 (223 mg, 77 µmol) was dissolved in dry THF (15 mL) and a solution of TBAF in THF was added (1M in THF, 383 µL, 383 µmol). The reaction mixture was stirred for 6.5 h at r.t. and then diluted with water. The aqueous phase was extracted with DCM. The combined organic phases were washed with water and brine and dried over $MgSO_4$. After removal of the solvent, the crude product was purified by recGPC to give 147 mg of **SI 12** as a yellow solid (74%).

Chemical formula: $C_{180}H_{274}O_{10}$, molecular weight: 2598.16 g mol^{-1}.

^{1}H-NMR (500 MHz, $CDCl_3$): δ [ppm] = 7.07 (s, 2H), 6.96 (s, 2H), 6.93 (s, 2H), 6.60 (d, ^4J = 2.2 Hz, 4H), 6.37 (t, ^4J = 2.2 Hz, 2H), 5.06 (s, 4H), 4.40 (s, 4H), 3.90-3.79 (m, 8H), 3.30 (s, 2H), 1.98-1.92 (m, 4H), 1.83-0.62 (m, 240H).

^{13}C-NMR (126 MHz, $CDCl_3$): δ [ppm] = 159.3, 154.7, 153.7, 153.6, 139.2, 118.1, 117.7, 116.7, 115.0, 114.7, 112.9, 106.3, 104.5, 91.7, 91.2, 82.4, 80.2, 72.4, 72.4, 72.3, 72.1, 71.6, 56.7, 56.5, 54.3, 42.8, 40.2, 39.7, 39.7, 39.6, 39.6, 36.4, 36.0, 35.9, 35.7, 32.9, 32.8, 32.1, 30.8, 30.7, 29.3, 29.3, 23.3, 29.3, 28.6, 28.4, 28.2, 25.8, 24.4, 24.1, 24.1, 24.1, 24.1, 23.2, 23.2, 23.0, 22.7, 20.9, 18.8, 14.3, 14.3, 12.2, 11.5, 11.4, 11.4, 11.4.

MS (MALDI TOF, DCTB): *m/z* (%) = 2597.1 (100) $[M]^{+\bullet}$.

GPC (PS-calibrated in THF): $M_p = 2.9\cdot10^3$ g mol^{-1}.

PPEB-3

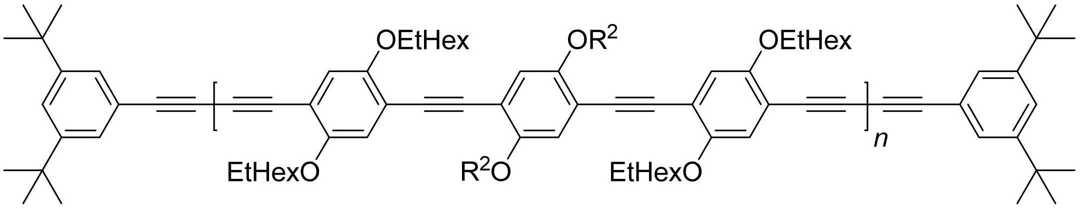

SI 12 (16 mg, 6.2 µmol) was dissolved in dry pyridine (2 mL) and purged with Ar for 10 min. CuCl (95 mg, 0.96 mmol) and $CuCl_2$ (19 mg, 0.14 mmol) were added and the resulting reaction mixture was stirred at r.t. for 140 min and 5 h at 50 °C. 1,3-Di-*tert*-butyl-5-ethinylbenzene[3] (5.3 mg, 24.7 µmol) was added and stirring at 50 °C continued for 15 h. The reaction mixture was diluted with water and the aqueous phase was extracted with DCM. The combined organic phases were washed with NH_3 (25%), 2M HCl, water and brine and dried over $MgSO_4$. After removal of the solvent, the crude product was purified by recGPC followed by filtration over silica (DCM) to give 15 mg of **PPEB-3** as a yellow solid. The product was separated into 10 fractions with low polydispersity via recGPC, from which one sample was investigated in detail ($M_n = 78.0\cdot10^3$ g mol^{-1} $M_p = 76.1\cdot10^3$ g mol^{-1}, $M_w = 83.3\cdot10^3$ g mol^{-1}, *PD* = 1.07).

Chemical formula: $C_{180}H_{272}O_{10}$ (repeating unit), $C_{212}H_{314}O_{10}$ (*n*=1), molecular weight: 2596.15 g mol^{-1} (repeating unit), 3022.76 g mol^{-1} (*n*=1).

^{1}H-NMR (500 MHz, $CDCl_3$): δ [ppm] = 7.07 (br s, 2H), 6.98 (br s, 2H), 6.94 (br s, 2H), 6.62 (br s, 4H), 6.38 (br s, 2H), 5.07 (br s, 4H), 4.42 (br s, 4H), 3.91-3.78 (m, 8H), 2.00-0.60 (m, 244H).

GPC of the crude polymer (PS-calibration in THF): $M_n = 13.0 \cdot 10^3$ g mol^{-1}, $M_p = 18.0 \cdot 10^3$ g mol^{-1}, $M_w = 24.7 \cdot 10^3$ g mol^{-1}, $PD = 1.90$.

Supplementary Table 2: GPC peak molecular weights, calculated molecular weights and mass peaks of **PPEB-3**.

Repeating unit *n*	GPC (PS-calibration) M_p (g mol^{-1})	calculation M (g mol^{-1})	MS (MALDI TOF, DCTB) *m/z* (%)
1	$3.1 \cdot 10^3$	3022.8	3022.3 (66) $[M]^{+\bullet}$
2	$6.0 \cdot 10^3$	5618.8	5868.6 (100) $[M+DCTB]^{+\bullet}$
3	$8.9 \cdot 10^3$	8214.9	8461.7 (100) $[M+DCTB]^{+\bullet}$

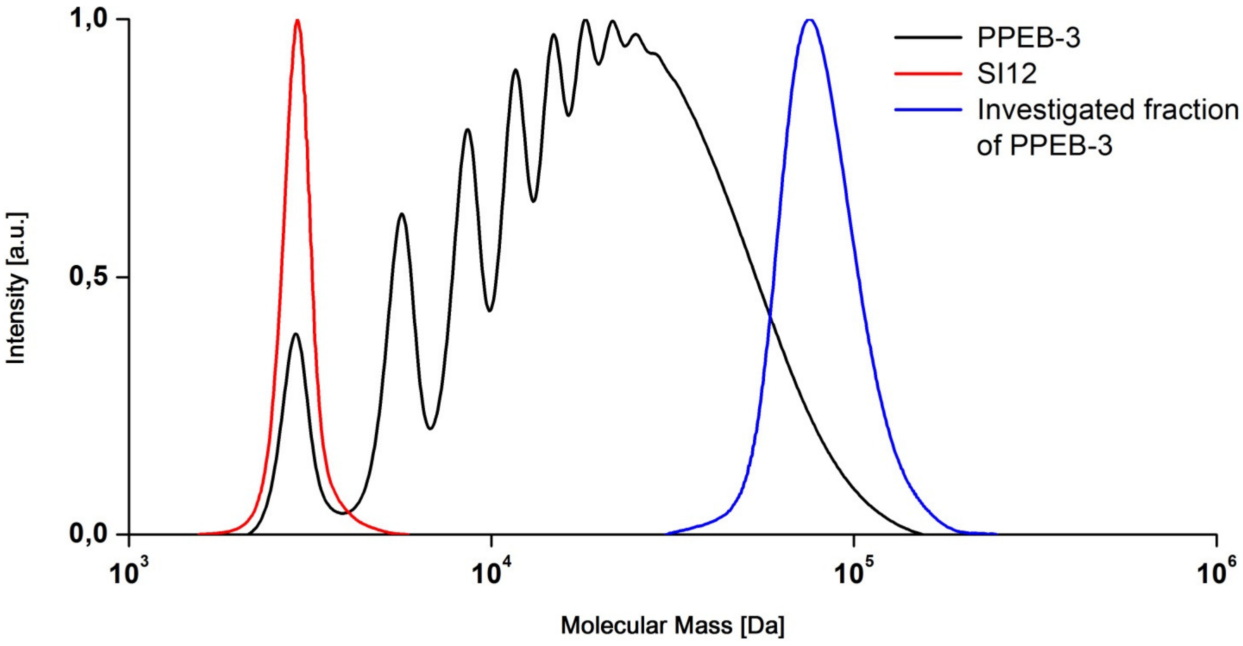

Supplementary Figure 6: GPC elugram of **PPEB-3** (black): $M_n = 13.0 \cdot 10^3$ g mol^{-1}, $M_p = 18.0 \cdot 10^3$ g mol^{-1}, $M_w = 24.7 \cdot 10^3$ g mol^{-1}, $PD = 1.90$; **SI12** (red): $M_n = 2.9 \cdot 10^3$ g mol^{-1}; $M_p = 2.9 \cdot 10^3$ g mol^{-1}, $M_w = 2.9 \cdot 10^3$ g mol^{-1}, $PD = 1.02$; **investigated fraction of PPEB-3** (blue): $M_n = 78.0 \cdot 10^3$ g mol^{-1}, $M_p = 76.1 \cdot 10^3$ g mol^{-1}, $M_w = 83.3 \cdot 10^3$ g mol^{-1}, $PD = 1.07$.

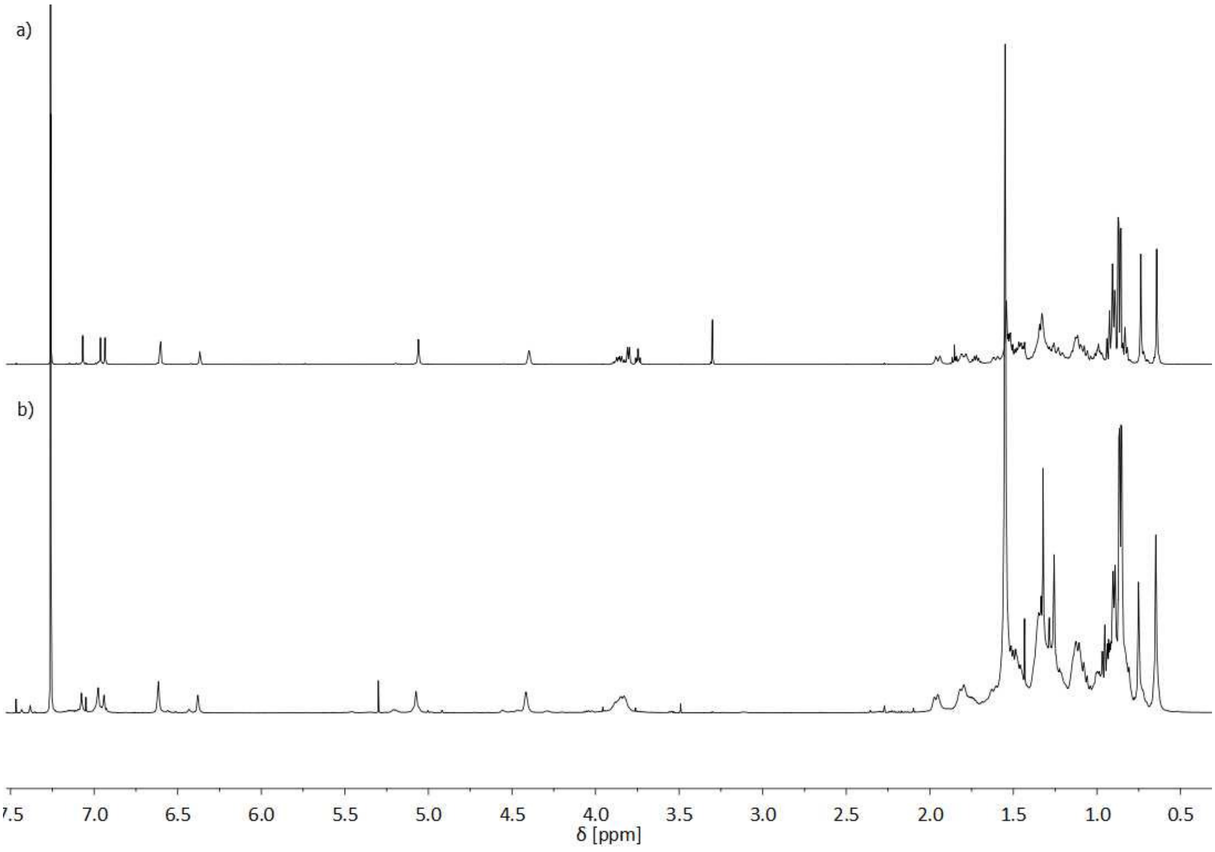

Supplementary Figure 7: a) ^{1}H-NMR spectrum (500 MHz, $CDCl_3$) of **SI 12**; b) ^{1}H-NMR spectrum (500 MHz, $CDCl_3$) of **PPEB-3**.

Supplementary References

1. Fulmer G. R., Miller A. J. M., Sherden N. H., Gottlieb H. E., Nudelman A., Stoltz B. M., Bercaw J. E., Goldberg K .I. NMR Chemical Shifts of Trace Impurities: Common Laboratory Solvents, Organics, and Gases in Deuterated Solvents Relevant to the Organometallic Chemist. *Organometallics* **29**, 2176-2179 (2010).

2. Jester S. S., Schmitz D., Eberhagen F., Höger S. Self-assembled monolayers of clamped oligo(phenylene-ethynylene-butadiynylene)s. *Chem. Commun.* **47**, 8838-8840 (2011).

3. Zhang R., Hao X., Li X., Zhou Z., Sun J., Cao R. Soluble Silver Acetylide for the Construction and Structural Conversion of All-Alkynyl-Stabilized High-Nuclearity Homoleptic Silver Clusters. *Cryst. Growth Des.* **15**, 2505-2513 (2015).

4. Watanabe K., Sun Z., Akagi K. Interchain Helically pi-Stacked Assembly of Cationic Chiral Poly(para-phenylene) Derivatives Enforced by Anionic pi-Conjugated Molecules through Both Electrostatic and pi-pi Interactions. *Chem. Mater.* **27**, 2895-2902 (2015).

5. Jester S. S., Shabelina N., Le Blanc S. M., Höger S. Oligomers and Cyclooligomers of Rigid Phenylene-Ethynylene-Butadiynylenes: Synthesis and Self-Assembled Monolayers. *Angew. Chem. Int. Ed.* **49**, 6101-6105 (2010).
